# Population Dynamics and Life History of *Euphorbia rosescens*, a Perennial Herb Endemic to Florida Scrub

**DOI:** 10.1371/journal.pone.0160014

**Published:** 2016-07-25

**Authors:** Stacy A. Smith, Eric S. Menges

**Affiliations:** Plant Ecology Program, Archbold Biological Station, Venus, Florida, United States of America; USDA-ARS, UNITED STATES

## Abstract

*Euphorbia rosescens* is a recently described plant that is narrowly endemic to the Lake Wales Ridge. Little is known of the ecology or life history of this diminutive, deeply rooted polygamodioecious perennial. We studied 13 subpopulations of this species from 2004–2012 from five habitats, sampling monthly during its growing season. Subpopulations were stable year-to-year with annual survivals > 90%, but with considerable within-year dynamics, peaking in density in April and dying back in the fall and winter. Stem densities did not vary among subpopulations, habitats, or by subpopulation gender. Annual plant dormancy was common and decreased subsequent survival. Belowground biomass averaged almost 50 times higher than aboveground biomass. Subpopulations either consisted of entirely female individuals or a mixture of male and functionally andromonoecious individuals and these subpopulation genders remained stable across years. Overall, flowering has been dominated by female plants. Plants produced modest numbers of inflorescences (cyathia), and fruit production was very low. Although most plants survived fire by resprouting, fire decreased survival and had a short-term positive effect on floral production. Lack of fecundity and recruitment are concerns for this state-endangered species, but more information is needed on its breeding system and clonality to make specific management recommendations.

## Introduction

Understanding population dynamics and general life history characteristics is a critical component of rare species conservation [1]. The production of management guidelines that will promote viable populations requires a fundamental knowledge of a species’ biology and the factors that drive population dynamics. Among the important factors that affect population persistence are population size, environmental stochasticity, breeding systems, genetic variation, plant and seed dormancy, habitat and microhabitat requirements, and geographic distributions [2, 3, 4, 5]. Demographic studies allow for the characterization of population dynamics in relation to habitat, weather, and land management activities and can provide the basis for effective conservation [6].

Understanding population dynamics and the factors that drive fluctuations and trends are clearly central to assessing endangerment and figuring out best management practices for rare species [7]. Longer lived plants with high annual survival generally tend to have populations that fluctuate little and may be able to better buffer environmental variation [8, 9]. Nonetheless, changes in drivers (such as climate change or alterations in a disturbance regime) may alter key vital rates (such as adult survival) and result in population declines [10, 11]. Even in these long-lived species, occasional recruitment will be necessary to balance low levels of mortality [12]. On the other side of the spectrum, short-lived plants often tend to have fluctuating populations and frequent extinctions [4]. Short-lived plants also tend to have high mortality rates and may need to continually recruit new members to the population to avoid declines [13]. When populations become very small, they are at risk of entering an extinction vortex, where interacting drivers may make extinction risk more severe [14].

One factor that may complicate estimates of population parameters is vegetative dormancy, which occurs when a plant remains alive belowground and does not produce any photosynthetic material for one or more growing seasons [15, 16]. Vegetative dormancy occurs in only a proportion of individuals within a population, unlike seasonal dormancy, where all individuals within a population go dormant. The occurrence of vegetative dormancy may have either negative or positive costs to survival [17] and when prolonged has been shown to have varied effects on fitness, particularly in the case of resource storage and assimilation [17, 18, 19, 20, 21] and reproduction [17, 22]. Additionally, the relationship between vegetative dormancy and life history traits is likely affected by the life span of the species [20].

Breeding systems can have various ecological effects on plant population dynamics and evolution. Flowering plants can exhibit a variety of sexual systems ranging from monoecism (pistillate and staminate flowers produced on same plant) to dioecism (pistillate and staminate flowers produced on the separate plants). However, several forms of sexual systems lie in between these two extremes, such as gynodioecy (pistillate and hermaphroditic flowers on different plants), androdioecy (staminate and hermaphroditic flowers on separate plants), and andromonoecy (staminate and hermaphrodite flowers on the same plant). Floral expression in these sexual polymorphic systems can be labile [23, 24] and have mixed evolutionary consequences on resource allocation, fitness, development [25], and ultimately reproductive output and recruitment [26].

Habitat and disturbance regimes may have large effects on population dynamics. Habitat can have significant effects on herbivory pressure [27], plant density [28], reproductive output [29] and seedling recruitment [30]. Changes within a habitat (i.e. disturbance) are important forces that shape and maintain the structure and function of an ecosystem [31, 32]. Fire is a disturbance that is shown to have strong effects on flowering phenology, recruitment, growth, and population persistence [33, 34, 35]. In fire-adapted habitats, increasing time-since-fire can lead to declines in some fire tolerant species [36, 37].

*Euphorbia rosescens* (Euphorbiaceae) is one of the least studied endemic plants on the Lake Wales Ridge. This narrow endemic is found only in Florida scrub, a fire maintained shrubland [38] that supports a wealth of endemic organisms [39, 40]. In this paper, we provide the first information on the ecology of this species. Specifically, we document demographic trends of *E*. *rosescens* across a variety of subpopulations, habitat types, and sexual morphs. We also documented plant responses to prescribed fire in a subset of subpopulations.

In addition to providing descriptive information on the biology and reproductive ecology of *E*. *rosescens*, we test several hypothesis based on the ecology of similar species occurring in Florida scrub, as well as general patterns found in the literature. We offer the following hypotheses:

1. Comparable to other resprouting Florida scrub species, individuals of *E*. *rosescens* will be long-lived and maintain high annual survival [41,42],
2. As shown in many other long lived perennial species, vegetative dormancy will have negative subsequent effects on survival and fecundity [43, 17];
3. Habitat disturbance along roadsides, by reducing competition from shrubs, will increase flowering and recruitment but decrease survival, as in other Florida scrub plants along roadsides [44].

## Material and Methods

### Study Species

Of the 2,000 *Euphorbia* species documented in the U.S., approximately 50 are found in the southeast coastal plain [45]. In Florida, 16 *Euphorbia* species have been documented [46]. In addition to being recently described [47], *E*. *rosescens* is one of three Euphorbia species listed as endangered in the state of Florida and one of five taxa within the section Tithymalus subsection Inundatae endemic to sandy ridges within the southeastern coastal plain. Of these five taxa, *E*. *rosescens* is the most narrowly endemic, with a range extending no greater than 50 km north to south, entirely confined to Highlands County in south-central Florida. *E*. *rosescens* is further restricted by habitat, occurring predominately on well drained white and gray soils within Florida scrub dominated by oaks and ericaceous shrubs and/or Florida rosemary [47]. Furthermore, extant populations of *E*. *rosescens* appear to be in decline, as only six of the 11 extant populations of *E*. *rosescens* have been confirmed as of 2002 [47].

Bridges and Orzell (2002), categorize *E*. *rosescens* taxonomically through morphological features. In its description, *E*. *rosescens* is characterized as a milky sapped perennial with vertical rootstock extending over 15 cm below-ground [47]. Since its discovery, *E*. *rosescens* belowground structure has been described as a xylopodium (Orzell personal communication) which consists of a lignified organ that exhibits both a shoot and root anatomical structure as well as having the capacity to form new shoot buds [48]. Such below-ground structures are often found in subtropical and tropical plant species and possibly evolved as an adaptation to fire and/or seasonal dry environments [48, 49].

Although primarily dioecious [47], *E*. *rosescens* has a polygamodioecious breeding system. Flowering in *Euphorbia* species occurs within cyathia arranged in a dichotomously branched inflorescence. Cyathia of *E*. *rosescens* are of three sex morphs: female, male or hermaphrodite. The co-occurrence of hermaphrodite and male cyathia on the same plant has been defined as functional andromonoecy and previously documented in 17 species of *Euphorbia* [50]. Populations either consist of entirely female plants or a mixture of male and functionally andromonoecious plants.

### Study site

Florida scrub is a xeric pyrogenic shrubland occurring throughout Florida and into parts of Georgia and Alabama [51, 52]. Several types of scrub can dominate xeric uplands, including sand pine scrub, rosemary scrub, scrubby flatwoods, oak-palmetto scrub, oak-hickory scrub, and (with fire suppression) xeric hammocks [52]. Across most types of Florida scrub, the dominant vegetation includes oaks, palmettos and ericaceous shrubs, whose composition and structure are shaped by infrequent high intensity fire [51, 53]. Tree cover (mainly *Pinus spp*.) varies with the type of scrub and time-since-fire. The groundcover layer includes a mixture of characteristic species such as gopher apple (*Licania michauxii*), milk peas (*Galactia spp*.), ground lichens (*Cladonia spp*.) and several endemics with limited distribution [51]. Most dominant shrubs and some perennial herbs retain most of their biomass belowground and resprout following fire [54, 55], however, many herbaceous plants are obligate seeders, some found mainly in gaps among shrubs [56, 57].

In Florida scrub, disturbances such as fire may have direct effects on vital rates such as survival, growth, and seed viability [58, 59, 60, 61, 62, 63]. Life history strategies in response to fire may vary by habitat and species with recovery strategies ranging from obligate seeding to resprouting from rootstock after plant material is consumed [52, 54]. In several scrub endemics, time-since-fire has critical effects on population persistence [64, 65, 66].

The Lake Wales Ridge, a geologic feature formed over one million years ago, is a documented biodiversity hotspot for a variety of endemic species [39, 40, 67]. This diversity is threatened by losses of habitat from development and agriculture [68]. Among the ecosystems on the Lake Wales Ridge, Florida scrub supports many narrowly endemic plant species [69]. A recent summary of the conservation status of the Florida scrub includes 36 endemic species, of which 28 are plants [40].

This study was conducted across three contiguous properties located in Highlands County, Florida, USA (27° 10’50”N, 81° 21’0”W). The first site is the main property of Archbold Biological Station (ABS) [70], a 2,101-ha private biological station that includes well-managed examples of xeromorphic Florida scrub [38, 53]. Florida scrub includes areas dominated by Florida rosemary as well as scrubby flatwoods, dominated by oaks and palmettos; both are habitats for *E*. *rosescens*. Sand roads traversing through scrub habitats provide additional habitat for *E*. *rosescens* and several other listed endemics. The second site, the Archbold Reserve, consists of native Florida scrub altered by agricultural activities (roller chopping, fire suppression, grazing) and exotic-grass dominated pastures still supporting an active cattle operation. We also studied the ecology of *E*. *rosescens* at the adjacent McJunkin property, a 120-ha tract of the Lake Wales Ridge Wildlife and Environmental Area managed by the Florida Fish and Wildlife Conservation Commission. This property is dominated by xeric scrub habitat degraded by a history of roller chopping and root raking prior to acquisition. Because all three properties are contiguous, we consider our study to have taken place within a single population of *E*. *rosescens*. We conducted our study among 13 subpopulations, defined as patches of stems separated from other subpopulations by at least 50 m.

### Excavation

In November 2004, we excavated a 2 m^2^ area (in a separate location from our demography subpopulations) containing 68 marked individuals to document belowground root lengths and determine if aboveground stems growing in close proximity to one another were connected by belowground rhizomes (i.e., whether the species was clonal). A trench was created, approximately two feet deep on two opposite sides of the plot, and the area was excavated horizontally from the trench. For each individual, we documented number of aboveground stems, aboveground stem length, and root length. Plants were dried at 70°C for 48 hours and above- and belowground parts were weighed separately.

### Demographic study

Over a series of years between 2004 and 2008, we subjectively selected study subpopulations to span the range of occupied habitats and representing (as possible) a range of time-since-fire from one to 40 years. In total we established 48 1m^2^ quadrats across 13 subpopulations in five habitat types (rosemary scrub, scrubby flatwoods, disturbed scrub, pasture, and roadsides; Table 1) and followed permanently marked individuals for nine years. Within each quadrat, we sampled each month from June 2004-June 2005. Subsequently, from 2006–2010, individuals were censused monthly from February through August. From 2011–2012 individuals were followed monthly from March through July. Each month we documented survival, number of stems, reproductive stage, and gender of each individual. Stem length (length of tallest stem for individuals with more than one stem) and reproductive effort (i.e. total number of cyathia) of each individual was recorded annually in June. Since cyathia develop throughout the flowering season, we estimated the total cyathia production by counting the bracteal leaf pairs subtending each cyathium. Closely occurring stems were assumed to be separate individuals if no root connections were found within the first few cm of the soil surface. We used plastic colored toothpicks, placed at the base of each stem, to follow separate individuals over time. We also documented recruitment, which was defined as including all newly observed untagged individuals. At no time did we observe cotyledons, thus all new recruits were considered new non-seedling adults, which probably re-emerged from dormancy or were products of clonal spread.

**Table 1:** Site, subpopulation number, number of quadrats, subpopulation gender, year quadrats were established, habitat, recent fire history, and number of individuals observed throughout the course of the study for 13 subpopulations of *E*. *rosescens* among three sites. All subpopulations are located on Satellite soils. ABS = Archbold Biological Station (West Section), Reserve = Archbold Reserve, LWRWEA = Lake Wales Ridge Wildlife and Environmental Area McJunkin Tract.

Site	Subpopulation	# Quads	Subpopulation gender	Year established	Habitat	Last burn	Total number of individuals observed
**ABS**	1	3	Mixed	2004	Roadside	n/a	98
**ABS**	2	2	Female	2004	Rosemary scrub	1993, 2010	141
**ABS**	4	2	Female	2005	Roadside	2010	116
**ABS**	5	2	Mixed	2005	Rosemary scrub	1993, 2010	65
**ABS**	6	4	Mixed	2005	Rosemary scrub	2004	56
**ABS**	9	3	Female	2005	Roadside	n/a	121
**ABS**	11	9	Mixed	2007	Scrubby flatwoods	1997	317
**ABS**	12	2	Mixed	2008	Rosemary scrub	1968, 2010	40
**ABS**	13	5	Mixed	2008	Rosemary scrub	1968, 2010	96
**Reserve**	3	3	Female	2004	Disturbed Rosemary scrub	unknown	115
**Reserve**	7	6	Female	2005	Disturbed Rosemary scrub	unknown	147
**Reserve**	10	2	Female	2006	Pasture	unknown, 2010	40
**LWRWEA**	8	5	Female	2005	Disturbed Rosemary scrub	n/a	111

Five study subpopulations were affected by prescribed fires in 2010. Four subpopulations burned in July (subpopulations 2, 4, 5, and 13) and one subpopulation (subpopulation 10) burned in February 2010. Within one month after the fire, we categorized fire severity for all individuals, scoring individuals as unburned, scorched (some leaf and stem material remained) or consumed (no aboveground material remained).

### Ethics Statement

All research was conducted on private (Archbold Biological Station) and public (Florida Fish and Wildlife Conservation Commission) land with the knowledge and permission from the landowners. The excavation of a single subpopulation took place at Archbold Biological Station prior to *E*. *rosescens* listing as endangered, thus we were not required to obtain a listed species harvest permit from the state of Florida, Department of Agriculture and Consumer Services, Division of Plant Industry. The individual in this manuscript has given written informed consent (as outlined in PLOS consent form) to publish these case details.

### Analysis

Stem abundances were determined by calculating the number of stems per square meter. Since plants were added over a series of years in this dataset we calculated annualized survival through 2012 by taking the proportion of cumulative survival and raising it to the power of the number of years. Both annual and annualized survival considered dormant individuals as alive.

Annual and seasonal dormancy were determined in subsequent censuses when a plant was recorded absent in a previous census and found alive in a following census. For the purpose of these analyses we did not use the last two years of data to avoid mistaking true mortality from annual dormancy.

Time-since-fire was calculated for each year (2005–2012) with a total of 17 time-since-fire classes ranging from 0 (year of fire) to 42 years since fire (classes include 0, 1, 2, 3, 4, 5, 6, 7, 8 12, 13, 14, 15, 16, 17, 41 and 42 years since fire).

In most cases we used nonparametric statistics due to difficulty meeting assumptions of parametric tests. Pearson Chi-Square tests were used to assess differences in annualized survival across habitats, annual flowering across years, annual dormancy effects on flowering, number of flowering events, and plant gender. Additionally, we used Pearson Chi-Square to test differences in recruitment by census year and habitat type, and fire severity and burn month effects on cumulative and annualized survival. Kruskal-Wallis tests were used to analyze variance in reproductive output across years. Mann-Whitney U tests were used to assess differences in reproductive output between subpopulation genders. Binary logistic regression was used to analyze habitat and subpopulation gender, and their interactive effects on flowering. We used the enter method and categorical data was set to simple contrasts. All data were analyzed in SPSS version 22. Figures were created in SigmaPlot version 11.0. Most analyses were done on data collected from 2004–2012. Because we added subpopulations throughout the study, analyses involving habitats were only possible from 2008 onward.

## Results

### Above- and belowground sizes

Aboveground, *E*. *rosescens* is a short statured herb, with stems rarely over 40 cm in length. Mean stem lengths ranged from 9.1 cm in 2006 and 2007 to 10.5 cm in 2005 and 2010 (median: 7.2 cm in 2007 to 8.4 cm in 2010). Among years, maximum stem length ranged from 33.4 cm in 2006 to 40.3 cm in 2010. Over the last four years of the study, stem lengths significantly varied among the five habitats (Kruskal-Wallis = 77.6 (2008), 105.2 (2009), 83.6 (2010), 65.9 (2011), and 98.5 (2012), p ≤ 0.001 in all years). Although pairwise differences were somewhat variable across years, there was a consistent annual trend of the greatest stem lengths within the pasture subpopulation and the lowest within scrubby flatwoods. In all years (2005–2012) stem length was significantly longer for flowering individuals (Mann-Whitney U > 38,810, p ≤ 0.001).

**Fig 1:**
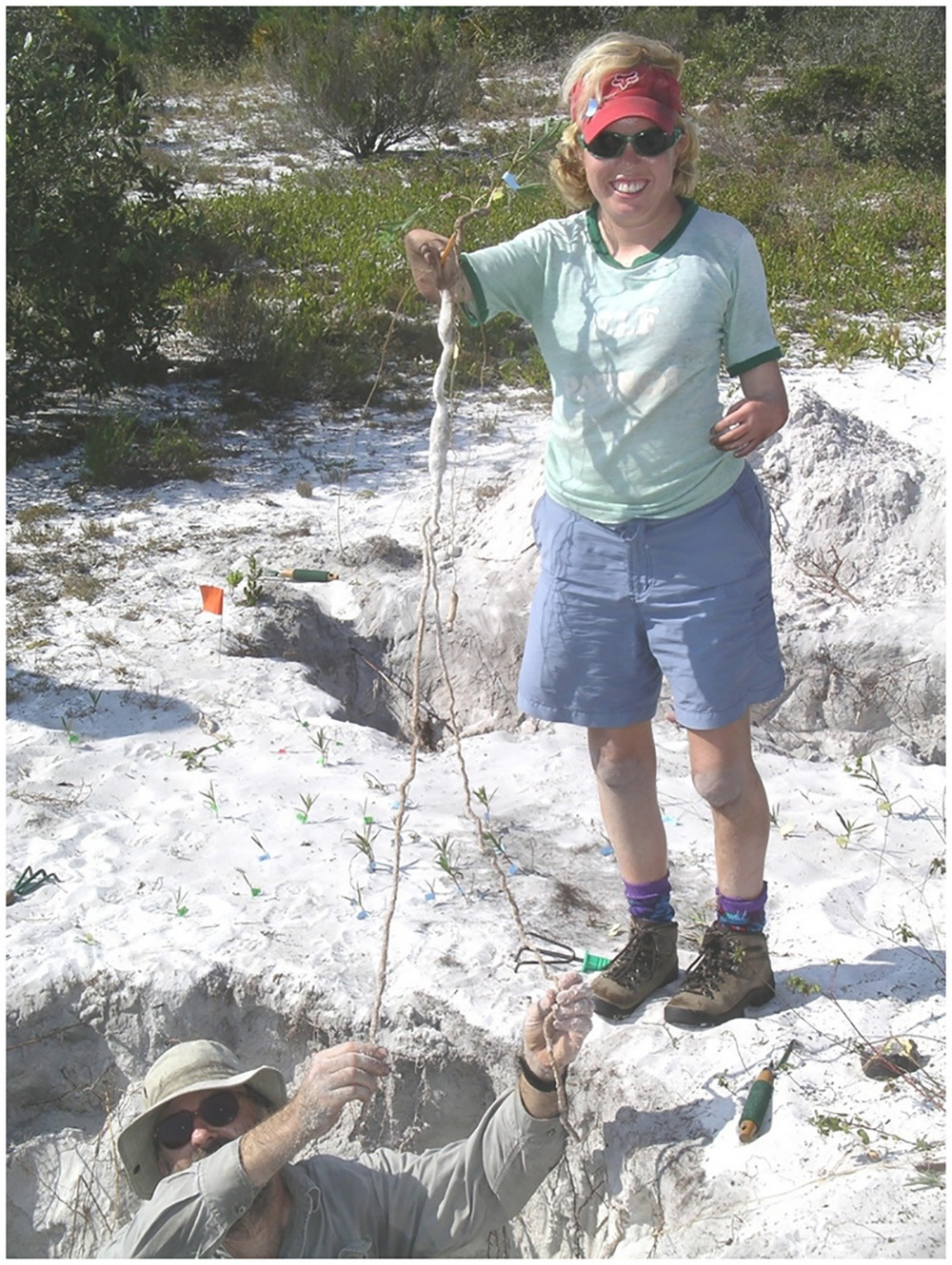
Archbold researchers Eric Menges (left) and Marcia Rickey (right) present an excavated individual of *E*. *rosescens*.

Our excavation showed that *E*. *rosescens* was deeply rooted and did not have belowground connections between stems (Fig 1). Of the 68 stems excavated, we were able to obtain partial belowground lengths on 52 and complete lengths on only ten. Intact roots were often relatively long (range 8.4–64.1 cm), while maximum aboveground length of excavated individuals only reached 10.0 cm (range 5.1–10.0 cm). Below- to aboveground biomass ratios were highly variable and had a median of 21:1. Of the 42 severed roots, below- to aboveground biomass ratios had a median of 16:1; these numbers underestimate the contribution of belowground biomass. Only one individual in the excavation area consisted of multiple stems, all of which (n = 3) were within 3 cm of each other and connected near the soil surface.

### Stem Abundance and Survival

**Fig 2:**
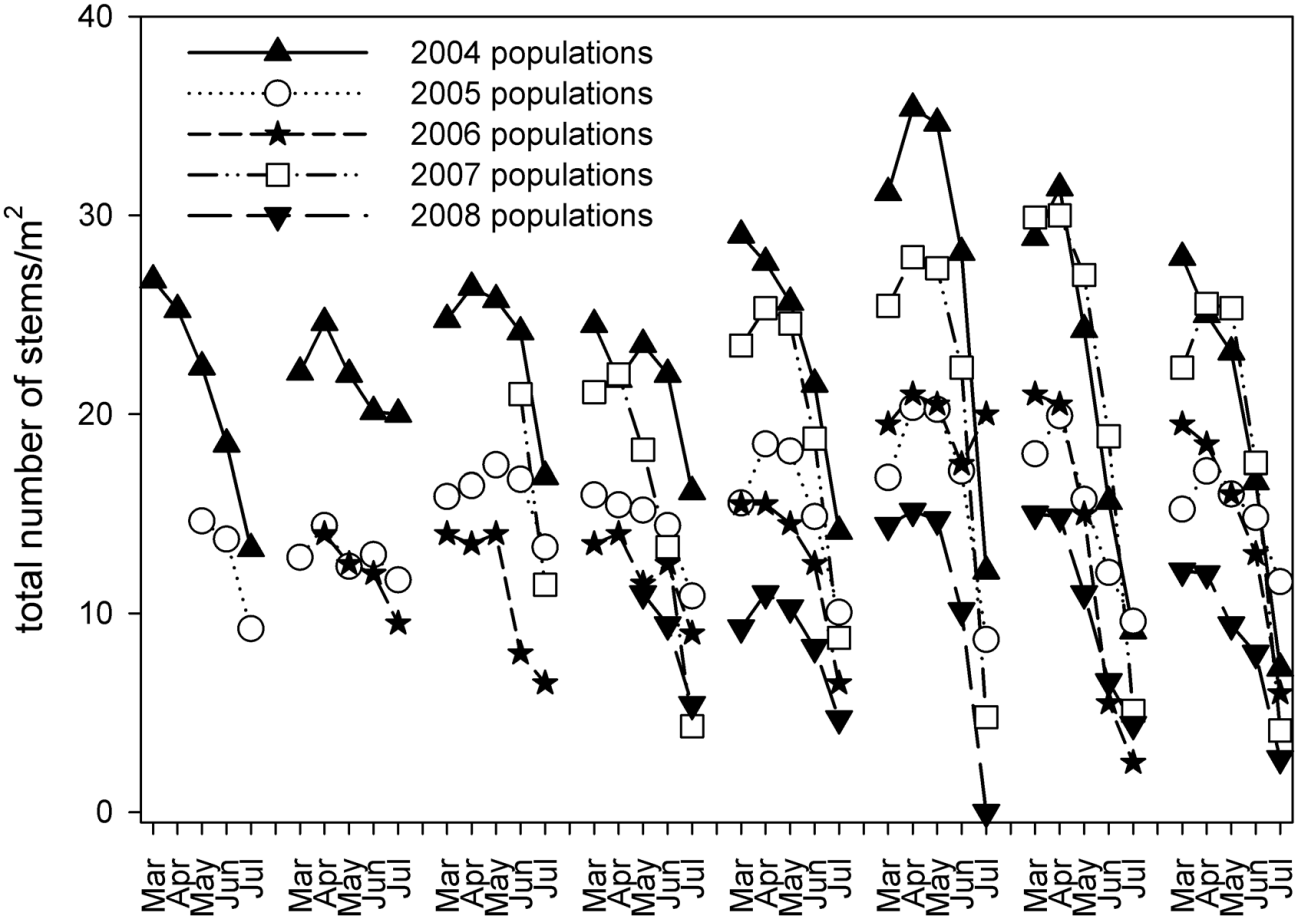
Monthly subpopulation densities for five groups of subpopulations (aggregated from the total of 13 subpopulations, based on the year we began research) of *Euphorbia rosescens* at Archbold Biological Station’s West Section, the Archbold Reserve, and Lake Wales Ridge Wildlife and Environmental Area McJunkin Unit from March 2005 through July 2012.

In our long-term demographic study, *E*. *rosescens* exhibited a consistent pattern of annual vegetative dormancy (Fig 2). For most individuals, aboveground stems died back between August and January. Average stem densities were highest in April (20 stems/m^2^) and lowest in August (8 stems/m^2^) across the years of this study.

The number of stems per individual ranged from one to four. Within each year (2005–2012), most individuals (> 90%) consisted of a single stem, while individuals with two or more stems were found much less frequently (<7%). Stem densities ranged from 22 to 26 per m^2^ across all study subpopulations between 2005 and 2012. Stem densities did not vary by subpopulation, gender, or habitat in any census year analyzed.

**Fig 3:**
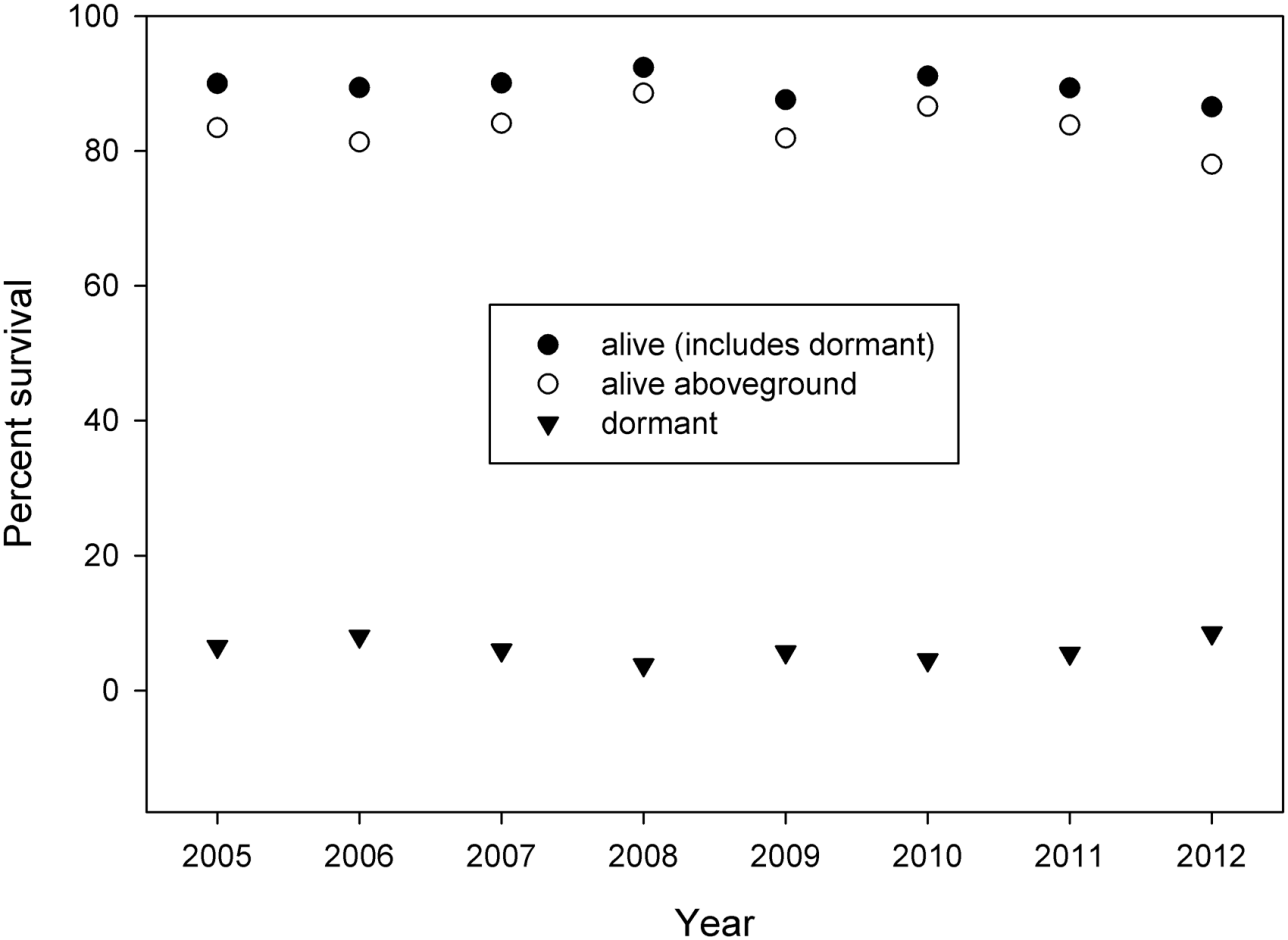
Annual survival of *E*. *rosescens* from 2005 to 2012. Percent survival is shown for three classifications: all live individuals including dormant plants, plants alive aboveground, and plants alive but dormant.

Overall, subpopulations of *E*. *rosescens* have remained stable or increased across census years. Average annual survival (including dormant individuals) across all study subpopulations was 89.6% with little variation (SD = 1.73) and mean annualized survival to 2012 was 91.6%. Within each year, we observed a mean of 83.5% (SD = 3.0) of individuals aboveground (Fig 3). Annualized survival significantly varied by habitat for most years from 2009–2012 (2009–10: Kruskal-Wallis = 0.033, p = 0.026; 2010–11: Kruskal-Wallis = 16.818, p = 0.002; 2011–12: Kruskal-Wallis = 14.712, p = 0.005). From 2009 to 2012, annualized survival was consistently higher among plants in rosemary scrub than disturbed scrub (2009–10: 99.0% vs. 90.7%, p = 0.026; 2010–11: 100% vs. 88.5%, p = 0.004; 2011–12: 100% vs. 90.5%, p = 0.005), although no other pairwise comparisons were significant.

### Annual Flowering

Subpopulations of *E*. *rosescens* are comprised of only two genders, female or a mix of male and bisexual plants, and subpopulations with different genders were spatially separated. Seven of our study subpopulations consisted entirely of female plants. Six subpopulations had a mixture of sexes, dominated primarily by male plants, with a smaller subset of individuals that were functionally andromonoecious (hermaphrodite and male cyathia on the same plant). At the subpopulation level, genders were stable across years (i.e. female subpopulations always dominated by female individuals).

Plant gender was unevenly distributed across the five habitat types (Table 1). While roadside and rosemary scrub habitats had at least one subpopulation with each gender, all disturbed scrub subpopulations consisted of female plants. The single subpopulation in scrubby flatwoods was mixed, while the pasture subpopulation was female.

Across all subpopulations we were able to identify the sex of >80% of flowering individuals in each year. Of those plants in which sex could be identified, flowering was dominated by female plants in all years. Across all subpopulations, within a given year, a mean of 75% of flowering plants were female, while an average of 21% of flowering plants were male, and 4% were functionally andromonoecious plants. Within mixed subpopulations, flowering across years among sexed individuals was dominated by male plants (mean 83%), with only 17% of flowering individuals being functionally andromonoecious.

**Fig 4:**
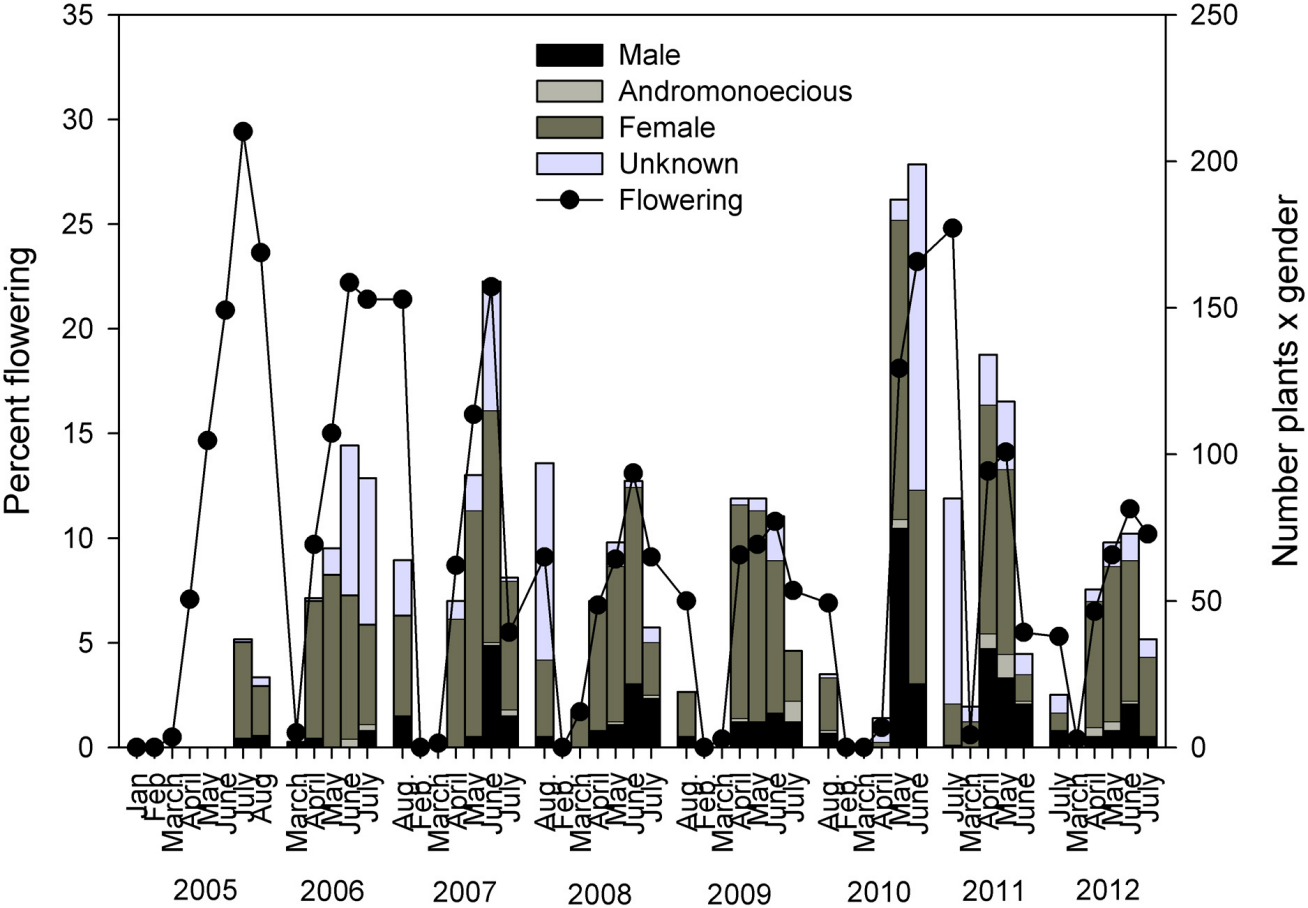
Percent flowering of *Euphorbia rosescens* plants (left axis, points and line) and number of male, andromonoecious (contain both male and hermaphrodite cyathia), or female plants (right axis bars) from February 2006 through July 2012. ‘Unknown’ indicates that plants were reproductive but sex could not be determined because cyathia abscised before flowers were observed.

Annual flowering among subpopulations always fell below 30% (Fig 4). Across all subpopulations, flowering was protracted, occurring in most years from March through August (Fig 4). Across years, mean annual flowering ranged from a low of 3% to a high of 39% and the proportion of flowering individuals varied significantly among subpopulations in all years of our study (for each year 2005–2012; χ^2^ > 44, df > 8, p ≤ 0.001). In most years, three female subpopulations (two disturbed subpopulations, 7 and 8 and one roadside subpopulation, 9) had the highest rates of flowering.

In all years, flowering varied significantly among habitats, with the highest percent flowering occurring in disturbed scrub in five of the eight years. In the remaining three years, flowering was highest for the pasture subpopulation (Table 2). In all but one year (2006) the proportion of flowering individuals varied significantly between the two subpopulation genders (Table 3), with a greater percentage of plants in female subpopulations in flower.

**Table 2:** Results of binary logistic regression of flowering in *E*. *rosescens* as a function of habitat and subpopulation gender (mixed vs. female) for each census year. Wald statistic, p value, Nagelkerke R and 2 LL values are given. Percent flowering is shown for each habitat type. Bold indicates pairwise significance between indicated habitats compared to rosemary scrub. Past. = Pasture, Scrub flat. = Scrubby flatwoods.

Year	Sig. predictor	Wald	p	Nag R	-2LL	Rose-mary	Road-side	Disturb scrub	Past.	Scrub flat.
**2005**	Gender° Habitat	3.743 7.400	0.053 0.025	0.060	461.756	5.0%	**11.4%**	**17.2%**		
**2006**	Habitat	13.556	≤0.004	0.038	769.065	13.2%	**24.9**%	**28.4**%	12.0%	
**2007**	Habitat	15.326	≤0.011	0.036	1004.672	14.1%	**25.0**%	**29.2**%	10.3%	**22.8**%
**2008**	Habitat	28.158	≤0.001	0.071	960.226	10.8%	**20.5**%	**23.9**%	**37.9**%	7.2%
**2009**	Habitat	19.749	≤0.030	0.090	935.212	12.4%	19.7%	**28.9**%	14.3%	**5.6**%
**2010**	Habitat	55.564	≤0.007	0.103	1040.225	8.5%	**5.0**%	**26.0**%	**69.0**%	**17.7**%
**2011**	Habitat	19.455	≤0.001	0.075	1046.072	11.3%	**24.1**%	**29.0**%	**42.9**%	12.1%
**2012**	Gender° Habitat	6.034 23.715	0.014 ≤0.005	0.108	806.834	4.7%	**15.5**%	**26.4**%	12.8%	**9.2**%

°greater proportion of plants in female subpopulations flowering

**Table 3:** Summary of flowering between subpopulation genders for each census year; for comparing flowering between genders, chi-square statistics (χ^2^) and significance (p) are presented.

Census year	% Flowering in mixed subpopulations	% Flowering in female subpopulations	χ^2^	P
**2005**	4.70%	14.20%	9.988	0.002
**2006**	17.50%	24.30%	3.368	0.066
**2007**	19.10%	25.40%	5.066	0.024
**2008**	11.20%	20.60%	18.237	≤0.001
**2009**	9.20%	23.10%	38.326	≤0.001
**2010**	15.90%	22.70%	8.424	0.004
**2011**	12.40%	25.70%	32.096	≤0.001
**2012**	5.10%	14.10%	34.647	≤0.001

### Plant Dormancy

While we have regularly documented a consistent pattern of intra-annual (i.e. seasonal) plant dormancy, *E*. *rosescens* is also subject to annual dormancy (i.e. vegetative dormancy where an individual is dormant ≥1 year). Since 2004, we have documented the occurrence of annual plant dormancy in 18% of the individuals in our dataset. Occurrence of annual dormancy ranged from one to three times over these nine years. Length of an annual dormancy episode ranged from one to seven consecutive years. Most individuals (68%) that experienced annual dormancy were only dormant for one year. Similarly, among plants experiencing more than one episode of annual dormancy, most (50%) were only dormant for one year at a time.

The occurrence of annual dormancy (i.e. plant dormant in any year between 2005 and 2012) significantly decreased subsequent annualized survival among our study plants (median of 95.0% versus 83.4% for plants experiencing annual dormancy (Mann-Whitney = 6.00, p = 0.05)). The number of annual dormancy episodes had no effect on annualized survival to 2012 (Kruskal-Wallis = 4.093, df = 6, p = 0.664; median annualized survival for one annual dormant episode = 83.4%, two = 89.2%, three = 87.2%, four = 97.5%, five = 0%, six = 0%, and seven = 45.9%), although plants experiencing fewer than four annual dormancy episodes had greater annualized survival than plants with five to seven episodes (>80% vs. <46%).

Annual dormancy did not significantly affect subsequent fecundity. Occurrence of annual dormancy had no significant effect on flowering (i.e., if plant ever flowered 2004–2012; 26.1% for dormant plants vs. 31.9% for plants never experiencing annual dormancy; χ^2^ = 3.472, df = 1, p = 0.062) or number of flower events (i.e. number of times a plant flowered from 2004–2012; χ^2^ = 15.086, df = 9, p = 0.089). Furthermore, the occurrence of annual dormancy was similar across plant genders (male: 17.1%, female: 15.0%, functionally andromonoecious: 14.3%; χ^2^ = 0.322, df = 2, p = 0.851) and between subpopulation genders (17.9% for both mixed and female subpopulations; χ^2^ = 0.002, df = 1, p = 0.968).

### Reproductive Output

Reproductive output (i.e., the number of cyathia, estimated by counting the remaining bracts that subtend each cyathium) was modest and affected by habitat but not plant gender. The total number of cyathia produced per plant averaged between six and 10. Reproductive output did not differ between subpopulation genders for any year of this study (2005–2012 Mann-Whitney U > 27, p > 0.181). However, in three of the five years (2008–2010), reproductive output varied significantly among habitats (Kruskal-Wallis ≥ 13.875, df = 4, p ≤ 0.008). Generally, in all years except 2012, reproductive output was highest in the disturbed scrub habitat. In pairwise tests, reproductive output was significantly greater in disturbed scrub compared to pasture and roadside subpopulations in 2008 (p ≤ 0.001), rosemary scrub subpopulations in 2009 (p = 0.008), and roadside subpopulations in 2010 (p = 0.004).

Fruit production was negligible during the course of this study. No fruit maturation was observed in any of our study subpopulations until 2011. From 2011 to 2012, we observed nine mature fruits among four subpopulations (three rosemary scrub subpopulations 2, 5, 13, and one scrubby flatwoods subpopulation 11).

### Recruitment

Despite not adding new sites or quadrats since 2008, we observed annual recruitment within all of our study subpopulations each year. Given low fruit production, these recruits were likely clonal recruits or plants emerging from annual dormancy. Although there was no significant difference between number recruits/m^2^ among years (Kruskal-Wallis = 7.00, df = 7, p = 0.429), recruitment did decline from 4.63 recruits/m^2^ in 2005 to only 1.58 recruits/m^2^ in 2012. New recruitment occurred mainly in the early spring, with most occurring in March (33.1%), followed by February (20.1%) then April (19.1%). The number of new recruits significantly varied among years (2005–2012; χ^2^ = 710.649, df = 7, p ≤ 0.001), with the highest recruitment occurring in 2006 and 2008.

Overall, the number of new recruits varied among subpopulations (χ^2^ = 89.707, df = 12, p ≤ 0.001) and habitat types (χ^2^ = 19.396, df = 4, p = 0.001). This trend was driven by pasture subpopulation 10, which produced the highest rates of recruitment (57.1%) across all study years. Subpopulations in disturbed scrub habitats had the lowest rates of recruitment (38.3%).

### Fire effects

Across the five subpopulations that burned in 2010, 67.4% (291) of individuals were affected by fire. Most affected individuals (62.2%) were consumed while 37.8% were scorched. Fire severity significantly affected cumulative survival to 2012 (χ^2^ = 8.196, df = 2, p = 0.017), with unburned plants having higher survival (81.2%), than scorched (79.4%) or consumed plants (69.1%). Burn month (February versus July) did not significantly affect subsequent survival (2010–2012; χ^2^ = 1.488, df = 1, p = 0.223).

**Fig 5:**
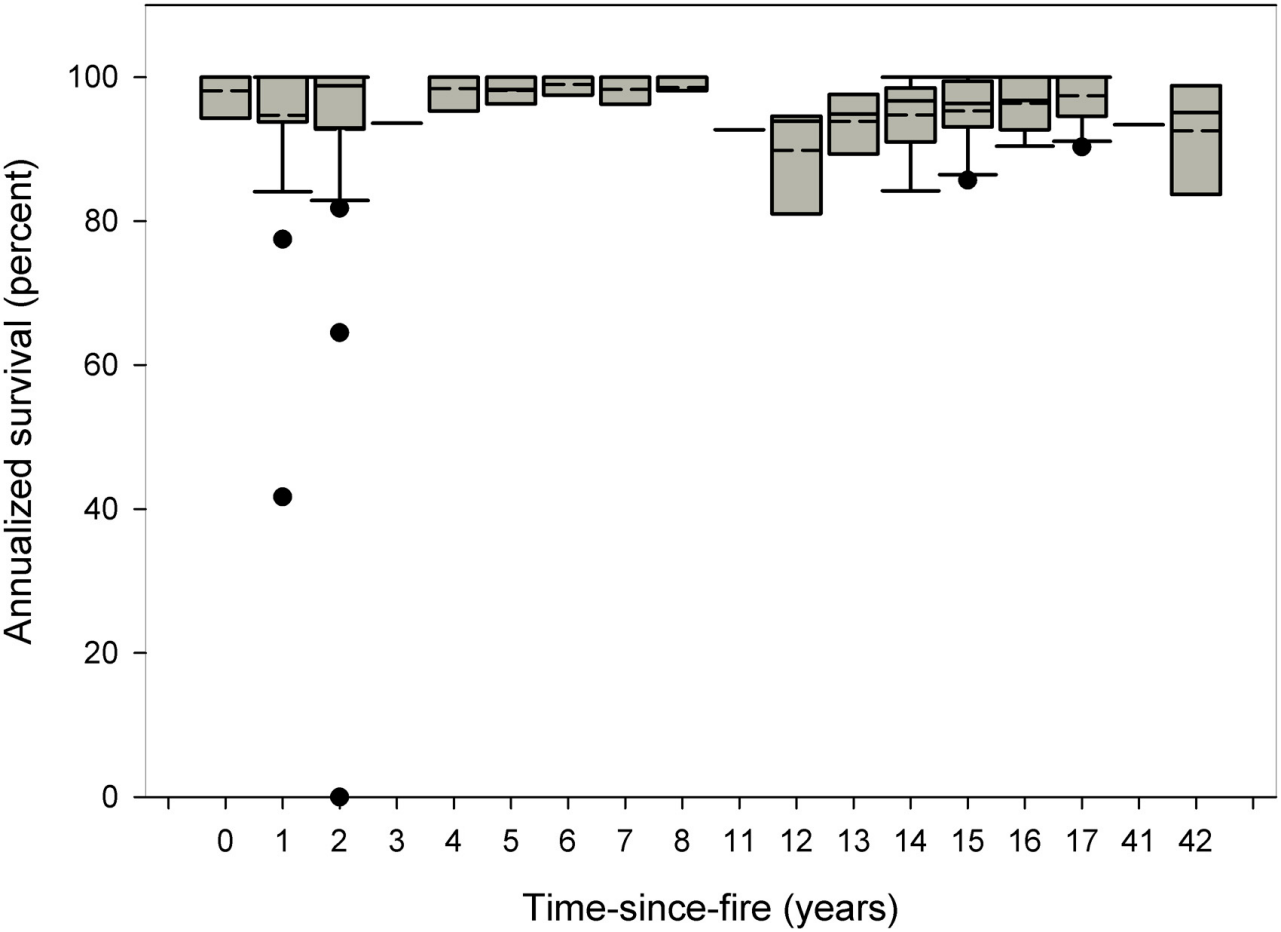
Annualized survival in relation to time-since-fire (in years) across eight subpopulations of *E*. *rosescens* for which fire history was known. Boxplots show mean (dashed line) and median (solid line). Black circles represent outliers. Sample sizes are included above each bar.

**Fig 6:**
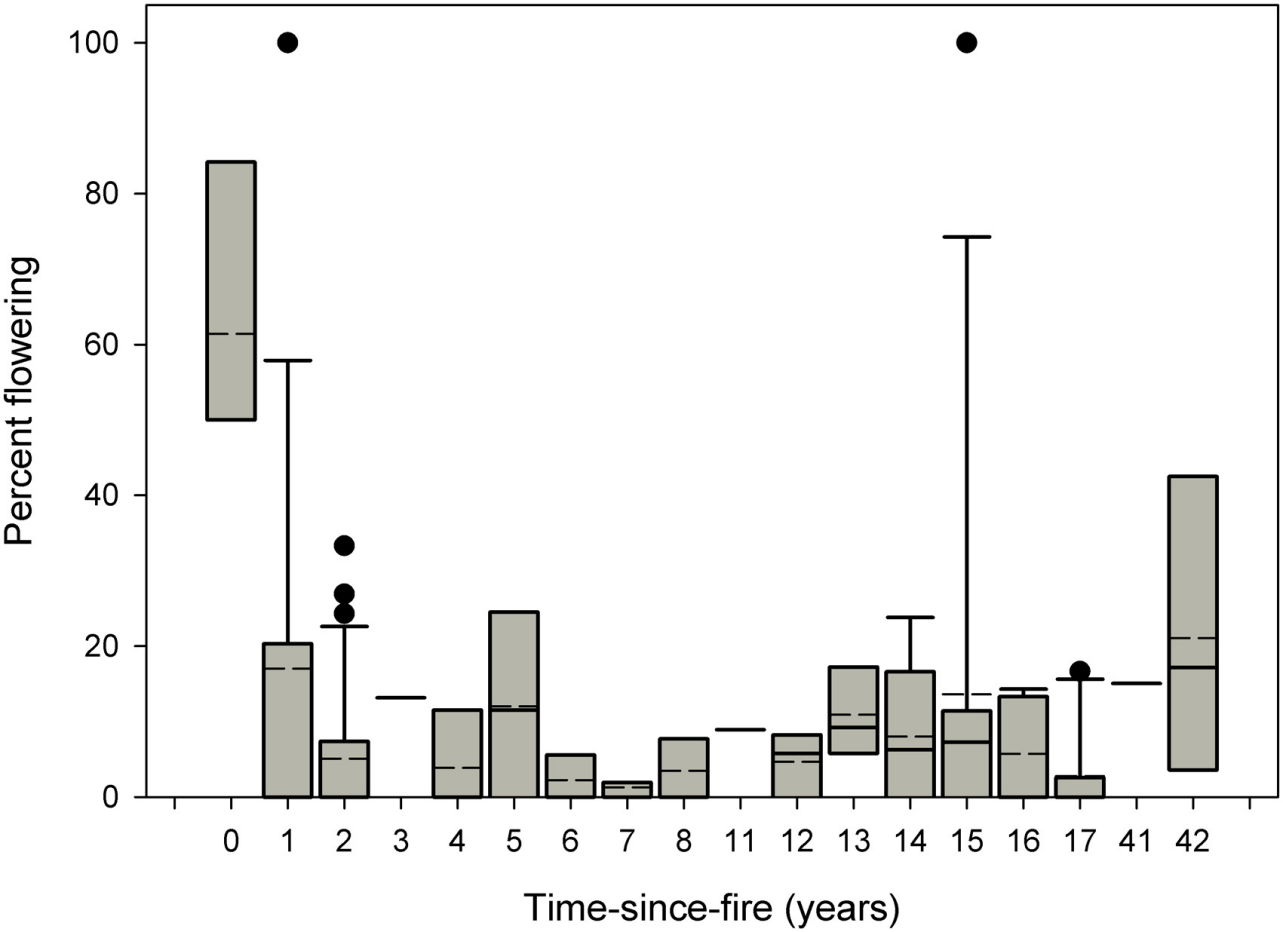
Percent flowering in relation to time-since-fire (in years) for eight subpopulations of *E*. *rosescens*. Boxplots show mean (dashed line) and median (solid line). Black circles represent outliers.

Annualized survival was unaffected by time-since-fire (Kruskal-Wallis = 19.278, df = 17, p = 0.313). In nearly all time-since-fire categories (range 0–42 years), annualized survival was >90% (Fig 5). Time-since-fire significantly affected flowering (Kruskal-Wallis = 30.404, df = 17, p = 0.024; Fig 6). Flowering was stimulated by fire, being markedly higher during the year of fire than in subsequent years (50% of plants flowering vs. <20% in other time-since-fire categories; Fig 6).

## Discussion

*E*. *rosescens* is a long-lived perennial with stable subpopulation dynamics across years. Average annual survival of most *E*. *rosescens* subpopulations was >90%; a high annual survival was hypothesized and is typical of many resprouting scrub species [41, 42]. *E*. *rosescens* is a small statured plant with extremely high root to shoot ratios; up to 49 times the amount of belowground biomass to aboveground biomass. This is more extreme, but similar to ratios up to 4:1 in long-lived clonal shrubs dominating Florida scrub [71, 72]. High root:shoot ratios may be advantageous for survival in drought-prone and nutrient-poor environments [73] as well as for dependable resprouting after annual dormancy. In Florida scrub, where soils are nutrient limited [74] and well drained, larger root systems may allow plants to tap directly into available ground water resources [75] as well as provide for more efficient exploitation of nutrients [76, 77].

All subpopulations of *E*. *rosescens* followed a consistent cycle of seasonal dormancy, where individuals were active aboveground during warmer months with seasonal rainfall, and dieback in the fall and winter. However, a small proportion (18%) of individuals were also subject to prolonged vegetative dormancy. Causes of vegetative dormancy have been linked to several environmental factors, including nutrient limitation [18, 43], herbivory [78], and drought [79]. Additionally, frequency and duration of vegetative dormancy has been linked to habitat, with plants in xeric habitats showing a greater proportion of dormancy and over longer periods of time compared to more mesic habitats [80]. Dormancy is not unprecedented among perennial species in the genus *Euphorbia* [81, 82] Contrary to our hypothesis, *E*. *rosescens’* annual dormancy had limited effects on survival and no effect on subsequent fecundity. Such results have been observed in previous studies, where Shefferson [43] found that vegetative dormancy in long-lived species was independent of survival and varied more with climatic factors.

As we hypothesized, habitat disturbance showed positive (increased flowering, reproductive output), negative (decreased annualized survival) and mixed (recruitment) effects on vital rates of *E*. *rosescens*. Although, we have to acknowledge the limitation of these inferences due to low samples sizes, such trends have been observed in other Florida scrub species, particularly herbaceous species specializing in gaps, or open spaces in the shrub matrix. For example, roadside populations of *Hypericum cumulicola*, a federally endangered herb, were found to have more variable life spans, faster growth and flowering and higher fecundity than scrub populations [44]. Populations of *Paronychia chartacea* ssp. *chartacea*, another federally listed endemic herb, showed higher survival and seed production in scrub, but greater recruitment along road edges [63]. *Liatris ohlingerae*, an herbaceous perennial, had higher survival and lower fecundity along roadsides compared to plants located in Florida scrub [83]. In a separate study, *Chamaecrista fasciculata*, an annual legume, showed greater population growth in degraded Florida scrub compared to intact Florida scrub [84].

While *E*. *rosescens* exhibits a polygamodioecious breeding system, overall flowering is dominated by female plants. In most years, floral output was similar between population genders but differed among habitats. In any case, cyathia production was modest and fruit production is intermittent. Although we do not know if *E*. *rosescens* is self-compatible, the genus *Euphorbia* is mainly considered a self-compatible group [85]. However, geitonogamous crosses have led to lower seed set in some species [85, 86], suggesting partial self-incompatibility. Self-incompatibility has been shown as the primary factor limiting fruit set in other rare long-lived Florida upland species such as *Ziziphus celata* [87] and *Prunus geniculata* [88]. Furthermore, low rates of flowering and fruit set have been documented in *Crotalaria avonensis*, an endangered herb found in the Florida scrub [89].

Despite limited fruit set, there was consistent annual recruitment in *E*. *rosescens*. It is likely that the appearance of new individuals is a result of older individuals cycling out of annual or seasonal dormancy, as the number of newly recruiting individuals decreased with time since quadrats were established. However, it is also possible that the primary reproductive strategy is to produce new stems by clonal growth and fragmentation, as the initial rhizomes may break down rapidly, a likelihood supported by documentation that individuals can reproduce through root fragmentation [90] as well as a recent study on spatial genetic patterns [91].

Flowering plants may exhibit a diversity of strategies for achieving both sexual and asexual reproduction. In the case of *E*. *rosescens*, clonal reproduction may provide several evolutionary benefits, even increasing fitness in the absence of sexual reproduction by reducing mortality and enabling expansion to new environments during times of environmental stress [92]. Furthermore, if sexual reproduction provides little contribution to fitness in *E*. *rosescens*, the vestigializaiton of sex may have occurred over a relatively short period of time, a process that has been documented in geographically peripheral populations of *Decodon verticillatus*, a clonal aquatic plant [93, 94]. However, clonal reproduction has its tradeoffs and may explain the imbalance in gender ratios across subpopulations used in our study, as clonality has been shown to disrupt morph ratios in dioecious species [95].

While the majority of *E*. *rosescens* plants resprouted post-fire, consumed plants had lower survival than unburned or lightly scorched plants. Although this could reflect fire-caused damage, it could also be related to pre- or post-fire conditions. Annualized survival was independent of time-since-fire. Flowering also showed no relation to time-since-fire except in the year of fire, where flowering percentages nearly tripled. Short-term fire responses have been observed among a variety of Florida scrub endemics [35, 37, 59, 61, 96] and may serve to promote delayed post-fire recruitment or replenish soil seed banks. Furthermore fire has positive effects on other southeastern *Euphorbia* species, such as *Euphorbia telephioides*, in which stem production was observed to increase post-fire [97].

### Management Implications

Lower survival following fire suggests this species prefers less frequent fires and may be favored by open gaps within Florida scrub habitats where fire effects are patchy, allowing plants to experience less frequent fires than the surrounding shrub matrix. Supporting this, *E*. *rosescens* was more likely to be found in open areas in a microhabitat study [98]. However, fire maintains and expands gaps in the habitats *E*. *rosescens* occupies [57, 99, 100], enhances *E*. *rosescens* flowering, and may ultimately increase fruit production in some subpopulations. For these reasons, fire may be the key to managing this imperiled species. However, there are not enough data to make specific recommendations on preferred fire return intervals at this time.

*E*. *rosescens* should be considered a globally imperiled species. Although high survival and stable subpopulation dynamics may compensate for low reproductive output, this narrowly endemic species occurs in a rare habitat threatened by historical clearing and fire suppression. Furthermore, only half of its extant populations have been confirmed within the past decade [47]. More research on its biology has the promise of providing useful management approaches to create viable populations.
